# In vitro determination of the protein quality of maize varieties cultivated in Malawi using the INFOGEST digestion method

**DOI:** 10.1016/j.heliyon.2023.e19797

**Published:** 2023-09-04

**Authors:** Molly Muleya, Dongfang Li, Gabriella Chiutsi-Phiri, Lester Botoman, John M. Brameld, Andrew M. Salter

**Affiliations:** a Future Food Beacon, University of Nottingham, Sutton Bonington Campus, Loughborough, Leicestershire, LE12 5RD, UK; b School of Biosciences, Division of Food, Nutrition and Dietetics, University of Nottingham, Sutton Bonington Campus, Loughborough, Leicestershire, LE12 5RD, UK; c Faculty of Life Science and Natural Resources, Natural Resources College, Lilongwe University of Agriculture and Natural Resources, P.O Box 143, Lilongwe, Malawi; d Department of Agricultural Research Services, Chitedze Agricultural Research Station, Lilongwe, Malawi

**Keywords:** DIAAS, Digestibility, Indispensable amino acids, Lysine, Quality protein maize (QPM), Zinc agronomically biofortified maize

## Abstract

There is an urgent need to alleviate protein deficiencies in low-income countries where cereal-based diets dominate. The objective of this study was to use the INFOGEST static digestion method and a recently established analytical workflow to determine the in vitro amino acid digestibility and protein quality of seven maize varieties grown in Malawi. Protein quality was measured using the in vitro digestible indispensable amino acid score (DIAAS). Amino acid digestibility was higher for the dehulled, low fibre, provitamin A maize flour (66%), compared to whole grain maize flours (51–61%), suggesting that the presence of fibre reduced digestibility (p < 0.05). Lysine was the limiting amino acid in all varieties, with the following DIAAS values for each variety; Provitamin A maize – 24, SC 719 – 32, Mtsikinya – 37, SC 167 – 39, Quality protein maize (QPM) – 40, Bantum – 40, SC 403 – 44. In addition to the variety of maize, protein quality was dependent on the level of processing and the agronomic practice applied with higher protein quality for the SC 403 variety in which zinc enriched fertilizer was applied. Comparing protein quality data with published in vivo data showed that DIAAS data were in closer agreement than amino acid digestibility data, which was slightly lower than published values, with mean in vitro amino acid digestibilities of 56–70% compared to a mean in vivo value of 77%. Overall, the in vitro method was able to correctly predict both the direction and magnitude of response. The INFOGEST digestion method coupled with the new analytical workflow will therefore be useful in the screening of high protein cereal crops and subsequent development of cereal-based foods with high protein quality.

## 1 Introduction

Cereal crops are an important source of energy for millions of people worldwide, with Maize (*Zea mays* L.*,* also known as corn) being particularly important in Latin America and sub-Saharan Africa (SSA) where it contributes at least 30% of food calories [1,2]. In SSA, as the population increases, the demand for maize is expected to triple by 2050 [3]. However, although maize is energy dense, it is not the best source of other nutrients, in particular proteins and micronutrients. Of note is the incomplete amino acid profile, being limited in the indispensable amino acids, lysine and tryptophan, associated with high protein deficiencies in regions where maize consumption dominates [4]. Protein and lysine deficiencies tend to dominate in the poorest individuals and households who have a poor diet quality, where maize is the main food source supplying the majority of macro and micronutrients. In Malawi, using a household consumption and expenditure survey (HCES), we showed that about 60% of the poorest households were at risk of lysine deficiency, based on apparent lysine intakes [5]. The risk of protein and lysine deficiency is likely to be exacerbated by the poor protein digestibility and utilizability of the maize staple.

The poor protein digestibility of maize, and other plant-based foods, is attributed to several factors. Similar to other plant based foods, maize contains anti-nutritional factors (ANFs) such as phytic acid and fibre that can restrict the digestion of proteins and absorption of amino acids [6]. In addition, the structural and cellular properties of the matrix, and the protein itself, are important determinants of the protein digestibility [7]. For example, the poor digestibility of the maize endosperm protein, zein, is due its hydrophobic nature, which causes the formation of water insoluble protein bodies that are inaccessible to digestive enzymes [8]. Our recently established database of in vivo ileal protein digestibilities [9] indicates that the protein digestibility of maize is around 82%. This means approximately 18% of protein in maize based diets is potentially unavailable and constitutes a nutritionally relevant loss, particularly in vulnerable segments of the population. For example, the risk of both protein and lysine deficiency in the poorest households of Malawi increased by 20% when gross apparent intakes were corrected for digestible intakes [5]. Utilizability is the ultimate measure of protein quality that indicates the efficiency to which the protein is absorbed and is based on both the digestibility and amino acid profile. Several methods have been used to measure protein quality, with the most commonly used being the protein digestibility corrected amino acid score (PDCAAS), which is slowly being replaced by the recently recommended digestible indispensable amino acid score (DIAAS) [10,11]. According to Ref. Moughan [12]; populations from low-income countries that depend on cereal protein, do not consume sufficient protein when gross protein supplies are corrected for utilizability using the DIAAS.

Considering the association of maize consumption with high levels of malnutrition in many low-income countries, improving the nutritional quality of maize, especially through biofortification, is recognized as a sustainable measure to address this challenge. Quality protein maize (QPM) is a group of biofortified maize varieties with enhanced levels of lysine and tryptophan, and a similar bioavailability to that of milk proteins [2,13]. Compared to common maize varieties, QPM varieties are suggested to accumulate at least 30 and 55% more lysine and tryptophan respectively [13]. This is due to the suppression of genes responsible for producing the lysine and tryptophan-deficient zein proteins in favour of the lysine and tryptophan-rich non-zein proteins [13,14]. Data on the actual lysine differential between QPM and common varieties is not clear, but is likely to be influenced by other parameters such as the local growing and environmental interactions. For example, protein contents of common maize varieties can range between 6 and 14% depending on the variety, location, planting rate, type and level of nitrogen fertilizer applied [[15], [16], [17]]. Accordingly, levels of lysine enhancement can also be expected to differ in different environments. Lysine enhancement levels of 10–58% and 30–82% have been reported for QPM varieties adapted to Canadian and Ethiopian environments, respectively [18,19]. Indeed Amegbor [15], showed significant variation in the protein and tryptophan contents of 26 QPM lines and 9 non-QPM lines grown in Zimbabwe and South Africa, with some non-QPM lines outperforming a few of the QPM lines in terms of their tryptophan content.

Clearly, locally relevant food composition data is needed to support any kind of nutritional intervention. In a previous study, we calculated that the risk of lysine deficiency in the poorest households in Malawi could be reduced by about 20% if QPM replaced the common maize varieties currently used in the diet, assuming a 30% increase in lysine composition of the QPM [5]. However, this simulation was not based on regionally relevant food composition data, as this was not available. Currently, data on the amino acid composition of foods is mainly derived from the USDA FoodData Central database, but this data is relevant for American products and not necessarily for other food systems, in particular for maize varieties consumed in Africa, which are distinctly different from those available in the USA [3]. In addition, although it is estimated that QPM has a superior digestibility to that of common maize, this is based on a relatively small number of studies [13] and has not been widely validated, particularly for the newer QPM varieties available in specific regions and in the form in which the maize is consumed by those populations. More importantly, the digestibility and DIAAS data for African maize varieties is not available due to the costs associated with the studies. Ileal digestibility is required to calculate DIAAS, and should preferably be determined in humans, growing pigs, or rats, in that order [10]. The constraints associated with current in vivo measurements necessitates the need for using an in vitro method that can be used to screen different types of food based on their amino acid digestibility and DIAAS. A new analytical workflow has recently been established [20], producing digestibility and DIAAS values that are in agreement with in vivo data, therefore providing a useful tool that can be used to rapidly assess the protein quality of foods and support the development of nutrition programs. The objective of this study was therefore to use the INFOGEST static digestion method, and the recently established analytical workflow, to determine the amino acid composition and digestibility, and overall protein quality of seven maize varieties grown in Malawi, including one QPM variety.

## 2 Materials and methods

All chemicals and reagents were purchased from Merck (Dorset, UK), unless stated otherwise. Grain samples of six maize varieties were obtained from Malawi in July 2021 (Table 1). The selected varieties are typically grown and consumed in Malawi except for the quality protein maize (QPM) which was obtained from Mgomera Seed Company, Lilongwe in Malawi and is still to be widely adopted. A commercial biofortified vitamin A maize flour was also obtained from a supermarket in Zambia in 2020. For each variety, the grains were cleaned of debris before milling, using a coffee grinder, to achieve a sieve size of <0.5 mm, which is the typical particle size for the flour used for cooking.

**Table 1:** Maize grain varieties included in the study.

Variety name	Description
QPM - Chitedze 2	Biofortified variety with enhanced levels of lysine and tryptophan, white
SC 719	Hybrid, white
SC 167	Hybrid, white
Bantum	Local variety, white
Mtsikinya	Local variety, yellow
Provitamin A	Biofortified with enhanced provitamin A levels, commercial sample, orange
SC 403	Agronomically biofortified with zinc, hybrid, white

QPM: Quality protein maize.

### 2.1 Determination of nitrogen content

Milled maize samples (later referred to as the ‘substrates’) were analysed for total nitrogen content using a nitrogen analyser (EA 1112 elemental analyser, Thermo Scientific, UK) following the Dumas method. A standard nitrogen-to-protein conversion factor of 6.25 was applied to calculate the protein contents. All measurements were conducted in triplicate for each sample.

### 2.2 In vitro digestion using the INFOGEST static digestion procedure

The milled maize samples (or substrates) were subjected to an in vitro digestion procedure according to Ref. Brodkorb [21] and following an optimised analytical workflow recently described by Ref. Sousa [20] with some modifications. For each maize sample, three independent in vitro digestions were performed on different days. A blank digestion comprising a ‘protein free cookie’ was included to account for the endogenous amino acids in the reagents (originating mainly from the enzymes) and to accurately mimic background digestion in the presence of food. The recipe for the protein free cookie was as follows: 40.8 g purified corn starch, 15.7 g sucrose, 4.9 g cellulose, 0.7 g baking powder, 0.5 g ginger, 36.9 g margarine. Additionally, a positive control of casein protein was also included and digested in parallel to the samples and the blanks. Casein was used as positive control as it is known to be highly digestible (digestibility >90%), which would be useful to indicate the success of the digestion. A food input weight of 1 g was used in which the amount of input protein was standardised to 8% or 80 mg protein for each sample digestion. The sample weight was adjusted to 1 g using MilliQ water (18.2 MΩ cm). For the oral phase, 1 mL of simulated salivary fluid (pH 7), containing CaCl_2_ (1.5 mM in final mixture) and α-amylase from Bacillus sp., (75 U/mL in final mixture) was added to 1 g of food. The mixture was incubated in a shaking water bath at 37 °C for 2 min. The gastric phase was followed by adding 2 mL of simulated gastric fluid comprising CaCl_2_ (0.15 mM in final mixture) and pepsin from porcine gastric mucosa (2000 U/mL in final mixture) and adjusting the pH to 3 using 2 M HCl. This mixture was incubated at 37 °C in a shaking water bath for 2 h, after which the pepsin activity was stopped by increasing the pH to 7 using 2 M NaOH. The intestinal phase of digestion was initiated by adding 4 mL of simulated intestinal fluid containing pancreatin from porcine pancreas (100 U/mL trypsin activity in final mixture), bovine bile (10 mM in final mixture) and CaCl_2_ (0.6 mM in final mixture). This mixture was incubated again for 2 h, at 37 °C in a shaking water bath. At the end of the intestinal phase, the tubes were immediately placed on ice, after which 32 mL of ice-cold absolute methanol was added followed by incubation at −20 °C overnight. An appropriate volume of methanol was added to achieve 80% (v/v) methanol in the final mixture. To separate the supernatants (containing absorbable peptides and free amino acids) and pellets (unabsorbable large peptides and undigested proteins), the samples were centrifuged at 4000×*g* for 15 min at 4 °C followed by carefully transferring the supernatants to new tubes and storing them at −20 °C until further analysis. The pellet was washed twice with 5 mL 80% methanol, with subsequent centrifugations after each wash (4000×*g*, 10 min, 4 °C) and each time discarding the wash solution. The tubes were left to stand in the fume hood for about an hour to evaporate the free methanol prior to snap freezing in liquid nitrogen, followed by freeze drying to constant weight. The dried pellets were stored at −20 °C pending further analysis. A mass balance was closely followed to account for the actual weights of the supernatants and the pellets, using the following equations:

>Equation 1 Weightofsupernatant(g)=W2−W3

>Equation 2 Weightofpellet(g)=W3−W1

where W1 is the weight of the empty tube before in vitro digestion (g), W2 is the weight of the digesta plus methanol (g) and W3 is the weight of the dried pellet after freeze drying (g).

### 2.3 Acid hydrolysis of substrates, digesta supernatants and pellets

To determine protein quality, the gross indispensable amino acid composition of the substrates and the associated amino acid digestibility were determined. Accordingly, an acid hydrolysis procedure which breaks down proteins into free amino acids is required for the substrates, as well as the digesta fractions (supernatants and pellets). A standard reference material of soy flour (National Institute of Standards and Technology, Maryland, USA, SRM 3234) was analysed in parallel to validate the accuracy of the hydrolysis and the amino acid analysis. The acid hydrolysis procedure consisted of the following steps:

*Sample weighing*: An aliquot of 50 mg was weighed into hydrolysis headspace glass tubes for the substrates while for the pellets, the whole pellet was weighed into the same hydrolysis glass tubes. In the case of the supernatants, an aliquot of 500 μL was placed in 2 mL eppendorf tubes and dried in a vacuum concentrator (Savant Thermo Scientific SpeedVac SPD140DDA) for 2–6 h.

*Oxidation*: A solution of freshly prepared performic acid (10% hydrogen peroxide in formic acid) was added to all samples (2.5 mL for the substrates and pellets, 2 mL for the supernatants). The tubes were incubated for 16–18 h at 4 °C with the caps open, followed by the addition of 0.42 g sodium metabisulfite (Na_2_S_2_O_5_) to decompose the excess performic acid. The oxidation solution oxidises the sulphur containing amino acids into products that are heat stable i.e., methionine is converted to methionine sulfone while cysteine is converted to cysteic acid.

*Acid hydrolysis*: A 6 M HCl solution with 1% phenol (to prevent amino acid oxidation) was added to all tubes (0.5 mL) followed by addition of 12 M HCl (2.5 mL for the substrates and pellets, 2 mL for the supernatants) to bring the whole mixture to a concentration of 6 M. The tubes were capped tightly before the mixture was hydrolysed in an oven at 110 °C for 24 h. The tubes were left to cool for a few minutes, before carefully transferring the mixture to new tubes using 20 mM ammonium formate, pH 2.75 as rinse solution. The pH of the solutions were adjusted to 2.75 using 4 M ammonium formate, before making the volume up to 50 mL with 20 mM ammonium formate. This was followed by centrifugation at 3000×*g* for 10 min and collection of the supernatants which were subsequently passed through a 0.22 μm syringe filter. The supernatants were stored at −20 °C prior to amino acid analysis.

The acid hydrolysis process causes the deamidation of asparagine into aspartic acid and glutamine into glutamic acid [22]. This means the measured levels of aspartic acid and glutamic acid will also include asparagine and glutamine respectively. In addition, tryptophan is destroyed during the acid hydrolysis process. Hence a separate hydrolysis procedure was performed to determine tryptophan in the substrates only. To an aliquot of 50 mg sample, 4.2 M NaOH with 1% phenol (2.5 mL) was added and placed in the oven at 110 °C for 17 h. A similar procedure as the one described for the acid hydrolysis was followed to obtain the final extracts for amino acid analysis, with the use of 6 M HCl to bring the pH to 2.75 before completing the volume to 50 mL using 20 mM ammonium formate (pH 2.75).

### 2.4 Amino acid analysis of substrates, digesta supernatants and pellets

The filtered extracts obtained from the acid hydrolysis and alkaline hydrolysis for tryptophan were used for the amino acid analysis. An aliquot of 200 μL was dispensed into HPLC vials followed by 200 μL of an internal standard comprising of cell-free isotopically labelled (^13^C, ^15^N) target amino acids. An amino acid standard curve was generated using Supelco amino acid standard mix, to which l-cysteic acid and methionine sulfone were added at a similar concentration with the amino acid standard mix. Targeted amino acids in samples and standards were analysed at 30 °C using a Thermo Scientific™ Acclaim™ Trinity P1 mixed mode column (150 mm × 2.1 mm, 3 μM) on a Thermo-Fisher Vanquish (uHPLC) coupled to an Altis Triple Quadrupole Mass spectrometer (MS/MS) with heated electrospray ionization (H-ESI) system. The amino acids were detected by multiple reaction monitoring (MRM) mode using positive electrospray ionization. A programmed gradient elution chromatographic separation (Table 2) was followed after injection of 1 μL of sample or standard.

**Table 2:** UHPLC gradient elution programme followed.

Time (min)	Flow rate (ml/min)	Mobile phase A (%)	Mobile Phase B (%)
0	0.3	100	0
5	0.3	100	0
7	0.3	0	100
14	0.3	0	100
14.5	0.35	100	0
16.5	0.35	100	0
17	0.3	100	0
18	0.3	100	0

Mobile phase A: 20 mM ammonium formate in water (pH 2.75) and mobile phase B: 100 mM ammonium formate in water and acetonitrile (80: 20, v/v); flow rate 300 μL/min.

The source conditions were as follows: spray voltage 3500 V, spray current 63.4 μA, ion transfer tube temperature 325 °C, vaporizer temperature 370 °C, sheath gas 5.58 L/min, auxiliary gas 7.97 L/min, ion transfer tube DC 15 V, RF Lens Amplitude 47 V. Nitrogen gas was produced using a nitrogen generator (Genius NM32LA, Peak Scientific Instruments Ltd). For each amino acid transition of interest, the collision energies (CE) were optimised, and one transition was used as the quantifier. Quantification for all targeted amino acids was achieved using the concentration vs peak area ratio (the integrated peak area of the analyte relative to that of the internal standard). Data acquisition was performed using Thermo Xcalibur™ mass spectrometry data system and data was processed using Thermo Tracefinder™ 4.1 application. Due to the loss of asparagine and glutamine in the acid hydrolysis process, 17 amino acids could be analysed, including both indispensable amino acids (IAA): Cystine (Cys), Histidine (His), Isoleucine (Ile), Leucine (Leu), Lysine (Lys), Methionine (Met), Phenylalanine (Phe), Threonine (Thr), Tryptophan (Trp), Tyrosine (Tyr) and Valine (Val) and dispensable amino acids: Alanine (Ala), Arginine (Arg), Aspartic acid (Asp), Glutamic acid (Glu), Glycine (Gly), Proline (Pro) and Serine (Ser). Recovery of amino acids was calculated using the soy SRM yielding a total amino acid recovery of 93%. All the data was normalized in relation to the expected recovery of the SRM and gross amino acid compositions of the substrates were expressed as g/kg.

### 2.5 Calculation of amino acid digestibility and digestible indispensable amino acid score (DIAAS)

To calculate amino acid digestibility, the amount of individual amino acids (mg) in the digesta supernatants and pellets was calculated after accounting for dilution factors and the weights obtained in the mass balance (Equation 1 and 2). The following equation was used to calculate amino acid digestibility:

>Equation 3 $$Amino\;acid\;digestibility\;\%=\frac{Fs-Cs}{\left(Fs-Cs\right)+\max\left(0;Fp-Cp\right)}\;*\;100$$

where Fs is the food supernatant (mg), Cs is the cookie (blank) supernatant (mg), Fp is the food pellet (mg) and Cp is the cookie (blank) pellet (mg). The cookie refers to the protein free cookie or blank used to account for endogenous amino acids. If Cp > Fp, the value was truncated to 0. The total amino acid digestibility was also calculated by considering the sum of all measured amino acids in both the digesta supernatants and pellets and applying equation (3) to calculate the digestibility. DIAAS was calculated in two steps according to equations Equation 4, >Equation 5):

Equation 4 InvitroDIAA(mg/gprotein)=IAA(mg/gprotein)*invitrodigestibilitycoefficientofIAA

where DIAA is the digestible indispensable amino acid, IAA is the mg of indispensable amino acid per g of protein calculated based on the gross IAA and protein compositions of the substrates and the in vitro digestibility coefficient determined in equation (3). Since tryptophan was not measured in the digesta supernatants and pellets, the total amino acid digestibility was used as an estimate to calculate the amount of digestible tryptophan.

>Equation 5 $$In\;vitro\;DIAAR\;\left(\%\right)=\frac{In\;vitro\;DIAA\;\left(mg/g\;protein\right)}{IAA\;of\;reference\;protein\;\left(mg/g\;protein\right)}*100$$

where DIAAR is the digestible indispensable amino acid ratio and the reference protein refers to the scoring pattern of a 6 month to 3-year-old child, recommended by the Food and Agriculture Organisation of the United Nations for regulatory purposes. Other scoring patterns for adults and infants less than 6 months old can also be used [23]. DIAAS is obtained by considering the minimum DIAAR, which is a value that can range from 0 to >1, and specifying the amino acid with the lowest DIAAR indicating the limiting amino acid of the substrate.

### 2.6 Comparison with in vivo data

The in vitro IAA digestibility values and subsequent DIAAS values were compared with published in vivo data in which standardised ileal amino acid digestibility was determined in pigs. We used our database [9] to select the publications in which similar substrates with adequate descriptions were included to allow a close match. One publication was found with complete data for casein [24], while for the maize varieties, a mean digestibility was calculated based on two studies in which raw, yellow dent whole grain maize flour was used [25,26].

### 2.7 Statistical analysis

Statistical analysis were performed using XLSTAT, version 2022.4.1 (378) [27]. One-way analysis of variance (ANOVA) was used to compare means for gross protein and amino acid compositions, in vitro digestibilities and digestible indispensable amino acid (DIAA) compositions. Where appropriate, post-hoc analysis was done using Tukey's Honest Significant Difference. Statistical significance was accepted at p < 0.05.

## 3 Results and discussion

We applied a newly established in vitro method to evaluate the protein quality of seven maize varieties cultivated in Malawi, including a recently released commercial provitamin A maize flour and a QPM variety. Maize is a target for several nutrition programs due to its importance as a food source for millions of households in SSA. A new in vitro method to accurately and rapidly determine the protein quality of maize, and other important protein sources, will be crucial to accelerate the development and formulation of urgently needed high quality protein-rich foods.

### 3.1 Gross protein and amino acid composition

The gross amino acid composition of the 7 maize varieties varied widely (Table 3), with the protein content ranging from 8.3 to 12.6%, and the agronomically biofortified with zinc maize (SC 403) having the highest protein content. The crude protein content was measured indirectly as nitrogen and, as expected, it correlated to the total amino acids for most varieties, particularly those where the total amino acids equated to 93–100% of the total protein. The provitamin A maize flour had the lowest total amino acid content, but this was not reflected in the total protein content, and only accounted for about 86% of the total protein. This discrepancy demonstrates the challenges in using a universal nitrogen-to-protein conversion factor of 6.25, which is now generally accepted to overestimate the protein content of some plant sources [28]. The nitrogen-to-protein conversion factor of 6.25 was established on the basis that proteins contain approximately 16% nitrogen. However, there are differences in amino acid profiles and non-protein nitrogen levels in some foods that could render this conversion factor inaccurate. For example, there is clearly a difference in the nitrogen-to-protein conversion factor for whole grain maize flour vs refined flour, based on how the protein content matches the sum of amino acids. Total amino acids closely matched crude protein values in the six maize grains that were analysed as whole grain flour. However, the provitamin A maize flour was refined using a milling process in which the bran fraction was removed. Zein proteins are the major type of proteins found in the maize endosperm and are particularly low in the essential amino acids, lysine and tryptophan, which contain side chains with additional nitrogen that could modify the nitrogen-to-protein conversion factor substantially. According to Ref. Mariotti [28]; accurate estimates of nitrogen-to-protein conversion factors for most plant proteins are close to 5.6. This factor is probably more accurate for the refined provitamin A maize flour as the total amino acids constitute about 96% of crude protein based on this estimate. Therefore, comparisons of different samples based on crude protein content alone and using a universal nitrogen-to-protein conversion factor, should be undertaken with caution.

**Table 3:** Gross protein and amino acid composition of some Malawian maize varieties.

	QPM	SC 719	SC 167	Bantum	Mtsikinya	Provitamin A	SC 403	ANOVA p-value
Protein	95.3 ± 1.5^cd^	84.4 ± 6.9^d^	83.4 ± 3.2^d^	104 ± 2.1^bc^	109 ± 4.2^b^	95.0 ± 7.3^cd^	126 ± 2.4^a^	<0.001
Total AAs	88.9 ± 0.1^cd^	79.6 ± 1.8^d^	83.2 ± 5.6^d^	97.3 ± 2.1^c^	111 ± 4.1^b^	81.8 ± 1.7^d^	127 ± 4.8^a^	<0.001
**IAA**								
CYS^1^	1.36 ± 0.1^b^	0.96 ± 0.1^de^	1.12 ± 0.1^cd^	1.28 ± 0.1^bc^	1.33 ± 0.1^b^	0.92 ± 0.1^e^	1.61 ± 0.1^a^	<0.001
HIS	3.51 ± 0.1^b^	2.70 ± 0.1^cd^	3.00 ± 0.3^c^	3.45 ± 0.1^b^	3.61 ± 0.1^b^	2.58 ± 0.1^d^	4.23 ± 0.2^a^	<0.001
ILE	4.58 ± 0.2^c^	4.42 ± 0.1^c^	4.43 ± 0.3^c^	5.48 ± 0.1^b^	6.38 ± 0.3^a^	4.92 ± 0.1^bc^	6.78 ± 0.3^a^	<0.001
LEU	8.45 ± 0.4^c^	8.28 ± 0.2^c^	8.29 ± 0.6^c^	10.1 ± 0.4^b^	12.1 ± 0.5^a^	9.15 ± 0.2^bc^	12.6 ± 0.5^a^	<0.001
LYS	3.00 ± 0.2^b^	2.52 ± 0.1^bc^	2.79 ± 0.2^b^	2.82 ± 0.1^b^	2.96 ± 0.2^b^	1.92 ± 0.1^c^	5.01 ± 0.5^a^	<0.001
MET	1.44 ± 0.1^cd^	1.21 ± 0.1^d^	1.31 ± 0.2^cd^	1.91 ± 0.1^b^	2.09 ± 0.1^b^	1.50 ± 0.1^c^	2.68 ± 0.1^a^	<0.001
PHE	4.43 ± 0.1^d^	4.08 ± 0.1^d^	4.21 ± 0.4^d^	5.20 ± 0.2^c^	5.93 ± 0.3^b^	4.50 ± 0.1^d^	6.60 ± 0.3^a^	<0.001
THR	3.10 ± 0.1^cd^	2.74 ± 0.1^de^	2.80 ± 0.2^de^	3.24 ± 0.1^c^	3.70 ± 0.2^b^	2.58 ± 0.1^e^	4.85 ± 0.2^a^	<0.001
TYR^1^	2.41 ± 0.1^d^	2.22 ± 0.2^d^	2.34 ± 0.1^d^	2.96 ± 0.2^c^	3.60 ± 0.3^b^	2.47 ± 0.1^d^	4.21 ± 0.1^a^	<0.001
VAL	4.93 ± 0.3^c^	4.25 ± 0.1^c^	4.65 ± 0.2^c^	5.04 ± 0.2^bc^	6.01 ± 0.7^ab^	4.11 ± 0.1^c^	6.90 ± 0.5^a^	<0.001
TRP	0.75 ± 0.1^ab^	0.55 ± 0.1^cd^	0.57 ± 0.1^cd^	0.64 ± 0.1^bc^	0.58 ± 0.1^cd^	0.46 ± 0.1^d^	0.84 ± 0.1^a^	<0.001
Total IAA	37.5 ± 0.4^cd^	33.5 ± 0.6^d^	35.1 ± 2.2^d^	41.7 ± 1.1^c^	47.9 ± 2.4^b^	34.7 ± 0.5^d^	55.8 ± 2.3^a^	<0.001
% LYS	3.15 ± 0.3^bc^	2.99 ± 0.1^bc^	3.34 ± 0.2^b^	2.73 ± 0.1^c^	2.72 ± 0.2^c^	2.02 ± 0.1^d^	3.98 ± 0.4^a^	<0.001
% TRP	0.79 ± 0.1^a^	0.65 ± 0.1^ab^	0.68 ± 0.1^ab^	0.62 ± 0.1^bc^	0.53 ± 0.1^bc^	0.48 ± 0.1^c^	0.67 ± 0.1^ab^	<0.001
**DispensableAmino acids**								
ALA	6.03 ± 0.4^b^	5.62 ± 0.2^b^	5.73 ± 0.1^b^	6.79 ± 0.4^b^	8.21 ± 0.9^a^	6.12 ± 0.1^b^	8.81 ± 0.7^a^	<0.001
ARG	5.24 ± 0.3^b^	3.80 ± 0.1^cd^	4.43 ± 0.3^bc^	4.72 ± 0.6^b^	5.08 ± 0.2^b^	3.23 ± 0.2^d^	7.16 ± 0.5^a^	<0.001
ASP	7.60 ± 0.4^bc^	6.54 ± 0.1^cd^	6.86 ± 0.7^cd^	7.50 ± 0.2^bc^	8.58 ± 0.3^b^	6.29 ± 0.4^d^	11.7 ± 0.5^a^	<0.001
GLU	16.6 ± 0.5^d^	16.2 ± 0.4^d^	15.9 ± 1.5^d^	19.6 ± 0.4^bc^	21.6 ± 0.9^ab^	17.5 ± 0.3^cd^	22.9 ± 1.5^a^	<0.001
GLY	2.37 ± 0.1^bc^	1.97 ± 0.1^c^	2.23 ± 0.1^bc^	2.21 ± 0.2^bc^	2.58 ± 0.2^b^	1.88 ± 0.1^c^	3.13 ± 0.4^a^	<0.001
PRO	10.1 ± 0.1^bc^	8.78 ± 0.3^d^	9.65 ± 0.7^cd^	10.9 ± 0.2^b^	12.2 ± 0.5^a^	8.81 ± 0.3^d^	12.4 ± 0.5^a^	<0.001
SER	3.56 ± 0.1^cd^	3.22 ± 0.1^d^	3.28 ± 0.2^d^	3.92 ± 0.1^bc^	4.37 ± 0.2^b^	3.24 ± 0.2^d^	5.06 ± 0.3^a^	<0.001

Values are means (g/kg DM) ± standard deviations of 3 replicates. IAA: indispensable amino acids, Total AAs: Total amino acids, %LYS and TRP are calculated relative to the protein content. ^1^Cystine and tyrosine are conditionally indispensable amino acids whose precursors are methionine and phenylalanine respectively. Hence they have been included as part of the indispensable amino acids since cystine + methionine make up sulphur amino acids (SAA) and tyrosine + phenylalanine make up aromatic amino acids (AAA) both of which are included in the calculation for protein quality. Means with different superscript letters are significantly different from each other (p < 0.05, post-hoc Tukey's Honest Significant Difference test).

The levels of both indispensable and dispensable amino acids were generally in the order SC 403 > Mtsikinya > Local Bantum > QPM > SC 167 = SC 719 = Provitamin A. Although the QPM was expected to have a higher lysine content than the other common maize varieties, this was not the case, with lysine only being significantly higher than provitamin A maize, while it was significantly lower than the SC 403 which contained 66% more lysine. The lower lysine content of the provitamin A maize was expected because, as stated previously, this is a refined maize flour dominated by lysine-poor zein endosperm proteins. Interestingly, the tryptophan content of the QPM was significantly higher than the other maize varieties, except for the SC 403. The percentage of tryptophan in QPM relative to the protein content was 0.79% (Table 3), close to the 0.8% required to classify a maize variety as a QPM [29]. In terms of lysine content, QPM lines are expected to have at least 4% lysine, but this was not the case with the QPM variety used in this study, which had about 3.2% lysine. It is not clear why the expected target levels for tryptophan were achieved whereas those for lysine were not. The performance of QPM varieties under different environments has been tested in several studies, with evidence of some common maize hybrids outperforming QPM under certain conditions [15]. Lower protein and lysine contents have also been reported for QPM grown under low nitrogen conditions [30], which is typical for small holder farming systems in SSA.

The SC 403 consistently, and surprisingly, had higher protein and amino acids in all cases. This maize variety was obtained from a field trial testing the effect of applying zinc fertilizers to different types of soils on zinc accumulation in the grain. It is possible that application of zinc to soils also influences the uptake and accumulation of nitrogen in the grain. However, other parameters that were not tested, including fertilizer application levels, genotype and agro-ecology may also be important. Hence, the potential association between zinc fertilizer application and protein content requires further investigation. The wide variation in the IAA compositions of the maize varieties also demonstrates the need for national and sub-national Food Composition Tables (FCTs) which capture the different varieties of crops grown in each region. Clearly, differences in the percentages of IAA ranging from 3.3 to 5.5% are nutritionally significant and need to be accounted for in FCTs.

### 3.2 Amino acid digestibility

The amino acid digestibility of the maize samples was determined using a newly established workflow, in combination with the INFOGEST static digestion procedure [20]. Casein was used as a positive control in order to validate the success of the in vitro digestion. The mean amino acid digestibility of casein was >90% (98%) which is expected for dairy proteins and indicates that the in vitro digestion was successful. There was a much smaller variation in amino acid digestibilities across the 7 maize varieties with the total amino acid digestibility generally in the order Provitamin A > Bantum = Mtsikinya = QPM = SC 719 = SC 167 > SC 403 (Table 4).

**Table 4:** In vitro amino acid digestibility of some Malawian maize varieties.

	QPM	SC 719	SC 167	Bantum	Mtsikinya	Provitamin A	SC 403	ANOVA p-value
IAA								
HIS	48.3 ± 6	49.0 ± 2	49.0 ± 5	56.0 ± 4	54.8 ± 8	54.8 ± 6	46.0 ± 5	0.211
ILE	63.0 ± 9	70.3 ± 7	70.2 ± 6	77.0 ± 3	72.0 ± 8	75.6 ± 4	63.7 ± 3	0.094
LEU	51.0 ± 6^bc^	55.0 ± 2^bc^	54.7 ± 4^bc^	62.7 ± 3^ab^	58.6 ± 5^ab^	68.3 ± 4^a^	45.9 ± 6^c^	0.001
LYS	73.0 ± 13	60.8 ± 9	66.5 ± 7	82.9 ± 7	76.5 ± 11	68.9 ± 11	63.7 ± 5	0.134
MET	66.1 ± 3	64.9 ± 4	66.4 ± 6	69.6 ± 4	67.7 ± 9	72.7 ± 2	60.7 ± 4	0.191
PHE	62.8 ± 8^ab^	61.0 ± 3^ab^	62.5 ± 2^ab^	69.3 ± 1^a^	65.3 ± 6^a^	72.4 ± 4^a^	51.8 ± 3^b^	0.002
THR	69.0 ± 10	66.1 ± 6	70.1 ± 4	75.1 ± 4	72.0 ± 5	74.2 ± 3	62.5 ± 3	0.147
VAL	57.5 ± 11^ab^	62.4 ± 1^ab^	62.6 ± 3^ab^	68.1 ± 2^a^	62.5 ± 8^ab^	67.3 ± 4^a^	51.5 ± 1^b^	0.030
**Dispensable amino acids**								
ALA	60.2 ± 4^bc^	64.1 ± 4^bc^	62.8 ± 2^bc^	70.2 ± 1^ab^	66.1 ± 7^abc^	75.5 ± 3^a^	56.0 ± 3^c^	<0.001
ARG	72.8 ± 12	66.6 ± 6	68.4 ± 5	76.1 ± 5	76.0 ± 7	72.9 ± 9	58.9 ± 4	0.109
ASP	54.7 ± 9^ab^	40.4 ± 8^b^	45.3 ± 5^b^	49.0 ± 6^ab^	61.5 ± 9^a^	43.3 ± 1^b^	48.1 ± 6^ab^	0.006
GLU	48.4 ± 1	46.9 ± 5	46.0 ± 6	55.3 ± 7	55.6 ± 9	60.8 ± 8	46.8 ± 8	0.110
PRO	50.5 ± 3^bc^	52.7 ± 2^bc^	54.3 ± 2^ab^	57.8 ± 2^ab^	56.1 ± 6^ab^	63.6 ± 4^a^	43.8 ± 4^c^	0.001
SER	63.0 ± 8	60.4 ± 6	62.8 ± 5	68.2 ± 9	67.6 ± 8	70.2 ± 8	54.6 ± 7	0.209
Total AAs	55.5 ± 5^ab^	55.1 ± 3^ab^	55.8 ± 4^ab^	63.7 ± 5^ab^	61.2 ± 8^ab^	66.0 ± 6^a^	50.8 ± 5^b^	0.036
Mean	60.0 ± 8.7	58.6 ± 3.9	60.1 ± 4.2	67.0 ± 2.4	65.1 ± 9.0	67.2 ± 2.8	53.9 ± 3.9	0.050

Values are means (%) ± standard deviations of 3 replicates. IAA: Indispensable amino acids. Total AAs: Total amino acids. Digestibility for cystine, glycine and tyrosine could not be accurately measured due to low concentrations in the digesta supernatants which could not be reliably discriminated from the blank measurements. Digestibility of tryptophan was not determined. Means with different superscript letters are significantly different from each other (p < 0.05, post-hoc Tukey's Honest Significant Difference test).

The provitamin A maize had 3–23% higher total amino acid digestibility than other maize varieties, most likely due to the lower fibre content resulting from the dehulling process. It is well recognized that fibre modifies protein and amino acid digestibility via several mechanisms [31]. The mechanism that explains the lower digestibility of non-dehulled vs., dehulled maize, or high vs. low fibre maize, is likely to be associated with the low accessibility of digestive enzymes to proteins that are encapsulated within the fibre matrix. Variation in the amino acid digestibility of other maize varieties, particularly the significantly lower digestibility of the SC 403 compared to others, also demonstrates differences in other characteristics of the maize grain or flour. Indeed, it has been previously shown that the chemical and physicochemical characteristics of the endosperm and kernel of maize can modify the digestibility of proteins [32]. For example, hard endosperm maize, characterised by a thick protein matrix surrounded by polyhedric starch granules, tends to exhibit lower protein digestibility than soft or floury endosperm maize with a thin protein matrix encapsulated in spherical starch granules [33,34]. Maize varieties vary quite widely in their degree of endosperm hardness and this may apply to the maize varieties used in this study, which comprised some hard dent and flint maize types. Although this trait was not measured in this study, it could explain some of the variation in digestibility observed and warrants a more focused investigation on this aspect. The lower digestibility of the SC 403 probably relates to the higher fibre content. The SC 403 flour had a distinct cream colour compared to the other whole grain maize flours, which were white, which is characteristic of small sized grains that have a higher ratio of bran (cream): endosperm (white) compared to larger grains.

The next stage was to calculate the in vitro digestible amino acid (DIAA) compositions of the different maize varieties (Table 5). The total DIAA was highest for the Mtsikinya (local variety) and the SC 403 (agronomically biofortified with zinc hybrid variety), despite the latter having the lowest amino acid digestibility. The reverse was true for the provitamin A maize which had the highest amino acid digestibility but a low DIAA. The local varieties or landraces (Bantum and Mtsikinya) tended to have higher DIAA than the other hybrid varieties (SC 167 and SC 719).

**Table 5:** In vitro digestible amino acid composition (DIAA) of some Malawian maize varieties.

	QPM	SC719	SC167	Bantum	Mtsikinya	Provitamin A	SC 403	ANOVA p-value
IAA								
CYS^1^	0.76 ± 0.1^ab^	0.53 ± 0.1^c^	0.63 ± 0.1^bc^	0.82 ± 0.1^a^	0.81 ± 0.1^a^	0.61 ± 0.1^bc^	0.82 ± 0.1^a^	<0.001
HIS	1.70 ± 0.2^ab^	1.32 ± 0.1^b^	1.47 ± 0.1^ab^	1.93 ± 0.2^a^	1.98 ± 0.3^a^	1.42 ± 0.2^b^	1.95 ± 0.2^a^	0.001
ILE	2.88 ± 0.4^b^	3.11 ± 0.3^b^	3.11 ± 0.3^b^	4.22 ± 0.2^a^	4.59 ± 0.5^a^	3.72 ± 0.2^ab^	4.31 ± 0.2^a^	<0.001
LEU	4.31 ± 0.5^d^	4.55 ± 0.2^cd^	4.53 ± 0.3^cd^	6.36 ± 0.3^ab^	7.10 ± 0.6^a^	6.25 ± 0.4^ab^	5.81 ± 0.7^bc^	<0.001
LYS	2.19 ± 0.4^bc^	1.53 ± 0.2^cd^	1.86 ± 0.2^bcd^	2.34 ± 0.2^b^	2.27 ± 0.3^bc^	1.33 ± 0.2^d^	3.19 ± 0.2^a^	<0.001
MET	0.95 ± 0.1^de^	0.79 ± 0.1^e^	0.87 ± 0.1^de^	1.33 ± 0.1^bc^	1.42 ± 0.2^ab^	1.09 ± 0.1^cd^	1.63 ± 0.1^a^	<0.001
PHE	2.78 ± 0.3^bc^	2.49 ± 0.1^c^	2.63 ± 0.1^bc^	3.60 ± 0.1^a^	3.87 ± 0.4^a^	3.26 ± 0.2^ab^	3.42 ± 0.2^a^	<0.001
THR	2.14 ± 0.3^cd^	1.81 ± 0.2^d^	1.96 ± 0.1^cd^	2.43 ± 0.1^bc^	2.65 ± 0.2^ab^	1.92 ± 0.1^d^	3.03 ± 0.1^a^	<0.001
TYR^1^	1.34 ± 0.1^c^	1.22 ± 0.1^c^	1.30 ± 0.1^c^	1.88 ± 0.1^ab^	2.20 ± 0.3^a^	1.63 ± 0.1^bc^	2.14 ± 0.2^a^	<0.001
VAL	2.84 ± 0.5^bc^	2.65 ± 0.1^c^	2.91 ± 0.1^bc^	3.43 ± 0.1^abc^	3.76 ± 0.5^a^	2.76 ± 0.2^bc^	3.55 ± 0.1^ab^	0.001
TRP	0.42 ± 0.01^a^	0.30 ± 0.01^c^	0.32 ± 0.01^bc^	0.41 ± 0.01^ab^	0.36 ± 0.01^bc^	0.30 ± 0.01^c^	0.43 ± 0.02^a^	0.001
Total IAA	22.3 ± 3.0^c^	20.3 ± 1.2^c^	21.6 ± 1.4^c^	28.8 ± 1.3^ab^	31.0 ± 3.2^a^	24.3 ± 1.4^bc^	30.3 ± 1.9^a^	<0.001
**Dispensable amino acids**								
ALA	3.63 ± 0.2^c^	3.60 ± 0.2^c^	3.60 ± 0.1^c^	4.77 ± 0.1^ab^	5.43 ± 0.5^a^	4.62 ± 0.2^b^	4.94 ± 0.3^ab^	<0.001
ARG	3.81 ± 0.6^ab^	2.53 ± 0.2^bc^	3.03 ± 0.2^c^	3.59 ± 0.2^ab^	3.86 ± 0.3^ab^	2.35 ± 0.3^c^	4.22 ± 0.3^a^	<0.001
ASP	4.16 ± 0.5^bc^	2.65 ± 0.5^d^	3.11 ± 0.4^cd^	3.68 ± 0.3^cd^	5.28 ± 0.5^ab^	2.72 ± 0.1^d^	5.64 ± 0.7^a^	<0.001
GLU	8.03 ± 0.2^b^	7.61 ± 0.9^b^	7.33 ± 1.0^b^	10.8 ± 1.3^ab^	12.0 ± 2.0^a^	10.6 ± 1.4^ab^	10.7 ± 1.8^ab^	0.003
GLY	1.31 ± 0.1^ab^	1.09 ± 0.1^b^	1.24 ± 0.1^ab^	1.41 ± 0.1^ab^	1.58 ± 0.2^a^	1.24 ± 0.1^ab^	1.59 ± 0.2^a^	0.002
PRO	5.07 ± 0.3^c^	4.63 ± 0.2^c^	5.24 ± 0.2^bc^	6.29 ± 0.2^ab^	6.83 ± 0.8^a^	5.61 ± 0.4^bc^	5.44 ± 0.6^bc^	<0.001
SER	2.24 ± 0.3^abc^	1.95 ± 0.2^c^	2.06 ± 0.2^bc^	2.67 ± 0.4^abc^	2.95 ± 0.4^a^	2.27 ± 0.3^abc^	2.76 ± 0.3^ab^	0.005
Total AAs	49.3 ± 4.6^bc^	43.9 ± 2.8^c^	46.4 ± 3.7^c^	62.0 ± 4.5^ab^	67.6 ± 8.6^a^	53.9 ± 4.6^abc^	64.7 ± 6.3^a^	<0.001

Values are means (g/kg DM) ± standard deviations of 3 replicates. IAA: indispensable amino acids, Total AAs: Total amino acids. ^1^Cystine and tyrosine are conditionally indispensable amino acids whose precursors are methionine and phenylalanine respectively. Hence they have been included as part of the indispensable amino acids since cystine + methionine make up sulphur amino acids (SAA) and tyrosine + phenylalanine make up aromatic amino acids (AAA) both of which are included in the calculation for protein quality. Means with different superscript letters are significantly different from each other (p < 0.05, post-hoc Tukey's Honest Significant Difference test).

### 3.3 Digestible indispensable amino acid ratios and scores (DIAAR and DIAAS)

The digestible indispensable amino acid ratios and scores are presented in Table 6 for 2 age groups i.e., for an older child, adolescent or adult and for a 6 month to 3 year old child. For regulatory purposes, FAO recommends using the scoring pattern of a 6 month to 3 year old. The limiting amino acid for all 7 varieties was identified as lysine with DIAAS values ranging from 24 to 44% (for 6 month to 3 year old) and 29–53% (for an older child, adolescent or adult). As expected, tryptophan was the second limiting amino acid in all the maize varieties, except for the hybrid maize variety, SC 167 where it was also the first limiting amino acid together with lysine.

**Table 6:** In vitro digestible indispensable amino acids ratios and scores (DIAAR & DIAAS) of seven Malawian maize varieties.

	QPM	SC 719	SC 167	Bantum	Mtsikinya	Provitamin A	SC 403	Casein
Older child, adolescent, and adult^a^								
HIS	1.11	0.98	1.10	1.17	1.14	0.93	0.97	1.99
ILE	1.01	1.23	1.24	1.36	1.41	1.30	1.14	1.74
LEU	0.74	0.88	0.89	1.01	1.07	1.08	0.76	1.71
LYS	0.48	0.38	0.46	0.47	0.43	0.29	0.53	1.87
SAA	0.78	0.68	0.78	0.90	0.89	0.78	0.85	1.27
AAA	1.05	1.07	1.15	1.29	1.36	1.26	1.08	2.73
THR	0.90	0.86	0.94	0.94	0.97	0.81	0.96	1.53
VAL	0.74	0.79	0.87	0.83	0.86	0.73	0.71	1.19
TRP	0.66	0.48	0.50	0.65	0.56	0.48	0.68	
DIAAS, %	48 (Lys)	38 (Lys)	46 (Lys)	47 (Lys)	43 (Lys)	29 (Lys)	53 (Lys)	119 (Val)
**6 months to 3 years old**^b^								
HIS	0.89	0.78	0.88	0.93	0.91	0.74	0.77	1.59
ILE	0.95	1.15	1.17	1.27	1.32	1.22	1.07	1.63
LEU	0.69	0.82	0.82	0.93	0.99	1.00	0.70	1.58
LYS	0.40	0.32	0.39	0.40	0.37	0.24	0.44	1.58
SAA	0.66	0.58	0.66	0.77	0.76	0.66	0.72	1.08
AAA	0.83	0.85	0.91	1.02	1.07	0.99	0.85	2.15
THR	0.72	0.69	0.76	0.76	0.78	0.65	0.78	1.23
VAL	0.69	0.73	0.81	0.77	0.80	0.68	0.66	1.11
TRP	0.51	0.37	0.39	0.50	0.44	0.38	0.53	
DIAAS, %	40 (Lys)	32 (Lys)	39 (Lys,Trp)	40 (Lys)	37 (Lys)	24 (Lys)	44 (Lys)	108 (SAA)

SAA: sulphur amino acids (methionine + cysteine), AAA: aromatic amino acids (phenylalanine + tyrosine). First limiting amino acid is in parenthesis.

Crude protein values used in the calculation for DIAAS were calculated using nitrogen-to protein conversion factor of 6.25. Tryptophan content of casein was not determined.

QPM - Quality protein maize, PDCAAS – protein digestibility corrected amino acid score, DIAAS – digestible indispensable amino acid score, DIAA – digestible indispensable amino acids, DIAAR – digestible indispensable amino acid ratio, IAA – indispensable amino acids, SAA – sulphur amino acids, AAA – aromatic amino acids, SSA – Sub-Saharan Africa, FCTs – Food composition tables.

a IAA reference pattern for older child, adolescent and adult expressed as mg AA/g protein: histidine, 16, isoleucine, 30, leucine, 61, lysine 48, SAA, 23, AAA, 41, threonine, 25, tryptophan, 6.6, valine, 40.

b IAA reference pattern for 6 months to 3 years old: histidine,20, isoleucine, 32, leucine, 66, lysine, 57, SAA, 27, AAA, 52, threonine, 31, tryptophan, 8.5, valine 43.

The provitamin A maize had the lowest DIAAS, a result of the low lysine content, some of which was probably lost in the dehulling process and could not be compensated by the higher amino acid digestibility. Dehulling of grains is a commonly practiced process to increase palatability of grains and to improve nutrient digestibility, as ANFs in the bran fraction are also removed. However, some beneficial nutrients may also be concurrently lost and, in the case of the provitamin A maize, the loss of lysine is seemingly greater than the gain in digestibility. The reverse is true for the SC 403, which had the highest DIAAS despite having the lowest amino acid digestibility. DIAAS was calculated using crude protein content (with a nitrogen-to-protein conversion factor of 6.25) as recommended by FAO [10]. However, as discussed in section 3.1, in some cases, the nitrogen-to-protein conversion factor of 6.25 may not be suitable for all food matrices, as in the case for the provitamin A maize flour. If the true protein content of the provitamin A flour were used to calculate DIAAS instead of the crude protein content, the DIAAS (6 months–3 year olds) for this maize variety will increase from 24 to 28. Although it still remains lower than the rest of the maize varieties, it highlights the need to rethink the use of crude protein contents. The wide variation in the protein qualities of the maize varieties provide some insights into future research that would further the quest to develop high protein maize-based foods.

1. The high protein quality of SC 403 (agronomically biofortified with zinc hybrid variety) suggests that agronomic management, including agronomic biofortification, could significantly improve the utilizable protein content of maize.
2. The low protein quality of provitamin A maize suggests that processing may influence the protein availability in maize, such that the trade-offs between improved digestibility, losses in nutrients and improved acceptability need to be investigated further.
3. There is substantial variation in the protein quality of different varieties, which could reflect better adaptability of some varieties than others in terms of nitrogen accumulation.

**Fig. 1:**
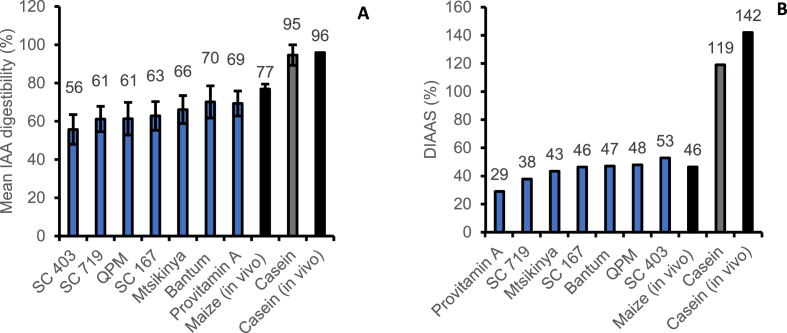
Comparison of mean IAA in vitro digestibilities and DIAAS with published in vivo values In vivo maize values are averages from 2 studies [25,26]. Casein in vivo data was obtained from Ref. [24].

If in vitro techniques are to be used to screen protein quality, it is critical that the measurements are in close agreement with in vivo values. This will be useful to accelerate the development of high protein quality foods based on accurate and reliable data. For this reason, we compared the mean IAA digestibility and DIAAS values derived in this study with the in vivo standardised ileal IAA digestibility and DIAAS values for maize derived from published studies which we had already compiled in a database [9]. Only two studies were found with adequate substrate descriptions that were considered to closely match the maize samples used in this study. The mean in vitro IAA digestibility for the maize varieties ranged from 56 to 70% (Fig. 1), which is close to a mean in vivo value of 77% for a yellow endosperm raw whole grain maize flour. Casein was used as a positive control in the in vitro digestions and the high mean IAA digestibility of 95% observed closely matches that of 96% observed in vivo. Likewise, the DIAAS values of 29–53% for a child, adolescents and adults closely agrees with a mean in vivo value of 46% for maize and accurately predicts lysine as the limiting amino acid.

Although the samples used in this study are not exactly the same as the samples used for comparison, the digestibility values are within a similar range, albeit slightly lower for the in vitro compared to the in vivo values. Indeed Sousa [20], showed a low mean difference of 1.2% when in vitro digestibility measurements were compared with in vivo values based on exactly the same test meals. The mean difference was even lower (0.1%) when comparing in vitro DIAAS with in vivo DIAAS, thereby demonstrating the accuracy of the method in predicting the protein quality of foods. The overall protein quality data indicates that use of the new analytical workflow integrated with the INFOGEST digestion method was able to predict the limiting amino acid and DIAAS with a good and acceptable degree of accuracy.

### 3.4 Methodological limitations

**Fig. 2:**
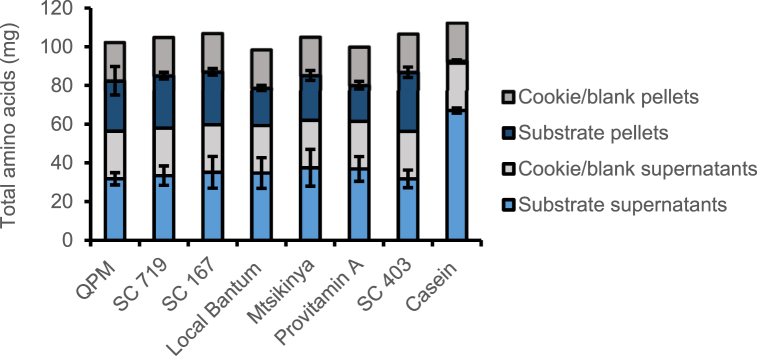
Total amino acids recovered from digestion of some Malawian maize varieties and casein.

Although the method used yielded amino acid digestibility results predicting the correct direction of response, some methodological challenges still exist which, if addressed, could further improve the accuracy and reliability of the measurements. The main challenge pertains to the substantial contribution of the endogenous amino acids (i.e., those from the enzymes) compared to sample amino acids. Fig. 2 clearly shows that endogenous amino acids contributed 40–50% of the total amino acids in the digesta, despite using 80 mg input sample protein compared to the recommended 40 mg protein [20]. This is not a problem in the case of highly digestible samples such as casein where, after in vitro digestion, most of the amino acids partition into the supernatant, resulting in a clear and large difference between sample and blank amino acids. In the case of lowly digestible samples, such as maize used in this study, the difference between supernatant amino acids in the samples and blanks is relatively low, potentially affecting the accuracy of the measurements. In that regard, we recommend using 80 mg protein for lowly digestible samples, and/or ensure that sample amino acids are at least 50% of total amino acids in the digesta to account for endogenous amino acids more reliably. More importantly, it is not clear whether the chemical and physicochemical behaviours of the endogenous amino acids and proteins are similar in all food matrices, particularly when dealing with food matrices with components that are known to interact/bind with proteins. For example, plant components in some cereals and legumes have been shown to interact differently with endogenous minerals derived from enzymes used in the INFOGEST digestion procedure [35]. Likewise, fibre components in some food matrices could bind to endogenous amino acids and proteins, potentially causing an underestimation of the amino acid digestibility when a universal blank correction is applied. The use of intrinsically labelled proteins could be useful to address this challenge.

As the amount of digested sample protein influences the protein digestibility, it is recommended to standardise the amount of input protein (i.e., 40 mg according to the original method). However, we decided to use 80 mg protein, in order to more clearly discriminate between sample and endogenous amino acids. However, dry matter contents are not standardised, meaning there could be large variations in the amount of dry matter digested, depending on the protein contents of the samples, and this could potentially modify the digestibility measurements. Variability in dry matter content is likely to impact on enzyme-substrate interactions. Lowly digestible food matrices are particularly challenging in this regard. Firstly, there is a greater likelihood of overestimating the digestibility of lowly digestible purified proteins, when low dry matter contents are required due to their high percentage of protein. This is likely due to the high enzyme-substrate interactions, increasing the chances of digestion. It has therefore been recommended to digest purified proteins in a food matrix mimicking the format in which they will be typically consumed [20]. Secondly, for low protein samples, with 2–8% protein, greater sample weight, sometimes even higher than the recommended input weight may be needed. This has the potential to underestimate digestibility due to the associated lower enzyme-substrate interactions. This could explain the slightly lower amino acid digestibilities of the maize samples used in this study compared to in vivo values. It also means that for samples requiring an input weight greater than the recommended, the volumes of simulated digestion fluids required for each phase of digestion may also need to be adjusted, which will add further complexity, particularly in handling the downstream processing after the in vitro digestion.

## 4 Conclusion

We have used for the first time, a recently established analytical workflow integrated with the INFOGEST static digestion procedure to determine the protein quality of seven maize varieties cultivated in Malawi. There was a wide variation in the total amino acid digestibility (51–66%) and DIAAS of the maize varieties, with lysine identified as the limiting amino acid (29–53% for a child, adolescents and adults and 24–44% for 6 months to 3 years old). An important and vital aspect of the method was the ability to show small variations across samples with subtle differences, thereby demonstrating the ability of the method to predict the correct direction of response. Clear differences in amino acid digestibility appeared to be related to the amount of fibre present, with whole grains having lower amino acid digestibility compared to a processed maize flour. However, other parameters such as agricultural management and variety were important in determining the overall protein quality. The in vitro amino acid digestibility and DIAAS values were in close agreement with published in vivo values in which whole grain maize flour was tested. Although some analytical challenges may still need to be resolved to improve accuracy, the new method is a useful screening tool to accelerate the identification and development of high quality protein crops and foods. For example, it could be used to shape breeding studies, agronomic interventions, and food processing for high protein crops and foods, as well as for screening of foods to be targeted for in vivo studies. This rapid, and relatively inexpensive, method potentially reduces our dependence on highly invasive and ethically challenging in vivo methods in humans or animals. Ultimately, it will enable the development of a comprehensive database for protein quality of foods in the form in which they are consumed. Such a database is urgently needed, particularly in regions such as SSA where population growth, and potential effects of climate change will make it increasingly challenging to meet protein requirements for populations where deficiency is already common. Such a database would be highly useful in the identification of local food sources that can be used to complement for example the lysine shortage in cereal-based diets in order to improve the protein quality of meals.

## Funding

This research was supported by the Future Food Beacon of the 10.13039/501100000837University of Nottingham. The funder had no role in the design of the study; in the collection, analysis and interpretation of data; in the writing of the report; and in the decision to submit the article for publication.

## Author contribution statement

Molly MULEYA: Conceived and designed the experiments; Performed the experiments; Analysed and interpreted the data; Contributed reagents, materials, analysis tools or data; Wrote the paper. Dongfang Li: Performed the experiments; Analysed and interpreted the data; Contributed reagents, materials, analysis tools or data. Gabriella Chiutsi-Phiri, Lester Botoman: Contributed reagents, materials, analysis tools or data. John M Brameld: Analysed and interpreted the data; Wrote the paper. Andrew M Salter: Conceived and designed the experiments; Analysed and interpreted the data; Wrote the paper.

## Data availability statement

Data included in article/supplementary material/referenced in article.

## Declaration of competing interest

The authors declare that they have no known competing financial interests or personal relationships that could have appeared to influence the work reported in this paper.

## References

[1] Erenstein O., Jaleta M., Sonder K., Mottaleb K., Prasanna B. (2022). Global maize production, consumption and trade: trends and R&D implications. Food Secur..

[2] Nuss E.T., Tanumihardjo S.A. (2011). Quality protein maize for Africa: closing the protein inadequacy gap in vulnerable populations. Adv. Nutr..

[3] Ekpa O., Palacios-Rojas N., Kruseman G., Fogliano V., Linnemann A.R. (2018). Sub-Saharan African maize-based foods: technological perspectives to increase the food and nutrition security impacts of maize breeding programmes. Global Food Secur..

[4] Schönfeldt H.C., Hall N.G. (2012). Dietary protein quality and malnutrition in Africa. Br. J. Nutr..

[5] Muleya M., Tang K., Broadley M.R., Salter A.M., Joy E.J. (2022). Limited supply of protein and lysine is prevalent among the poorest households in Malawi and exacerbated by low protein quality. Nutrients.

[6] Sá A.G.A., Moreno Y.M.F., Carciofi B.A.M. (2020). Food processing for the improvement of plant proteins digestibility. Crit. Rev. Food Sci. Nutr..

[7] Duijsens D., Gwala S., Pallares A.P., Pälchen K., Hendrickx M., Grauwet T. (2021). How postharvest variables in the pulse value chain affect nutrient digestibility and bioaccessibility. Compr. Rev. Food Sci. Food Saf..

[8] Calvez J., Benoit S., Piedcoq J., Khodorova N., Azzout-Marniche D., Tomé D., Gaudichon C. (2021). Very low ileal nitrogen and amino acid digestibility of zein compared to whey protein isolate in healthy volunteers. Am. J. Clin. Nutr..

[9] Muleya M., Salter A. (2021). Ileal amino acid digestibility and DIAAS values of world foods. Mendeley Data.

[10] FAO (2013). Dietary protein quality evaluation in human nutrition. FAO Food Nutr. Pap..

[11] Moughan P.J. (2019).

[12] Moughan P.J. (2021). Population protein intakes and food sustainability indices: the metrics matter. Global Food Secur..

[13] Prasanna B., Vasal S., Kassahun B., Singh N. (2001). Quality protein maize. Curr. Sci..

[14] Chand G., Muthusamy V., Allen T., Zunjare R.U., Mishra S.J., Singh B., Sarika K. (2022). Composition of lysine and tryptophan among biofortified-maize possessing novel combination of opaque2 and opaque16 genes. J. Food Compos. Anal..

[15] Amegbor I., Van Biljon A., Shargie N., Tarekegne A., Labuschagne M. (2022). Identifying quality protein maize inbred lines for improved nutritional value of maize in Southern Africa. Foods.

[16] Genter C., Eheart J., Linkous W. (1956). Effects of location, hybrid, fertilizer, and rate of planting on the oil and protein contents of corn grain 1. Agron. J..

[17] Ochieng I.O., Gitari H.I., Mochoge B., Rezaei-Chiyaneh E., Gweyi-Onyango J.P. (2021). Optimizing maize yield, nitrogen efficacy and grain protein content under different N forms and rates. J. Soil Sci. Plant Nutr..

[18] Fufa H., Akalu G., Wondimu A., Taffesse S., Gebre T., Schlosser K., Henle T. (2003). Assessment of protein nutritional quality and effects of traditional processes: a comparison between Ethiopian quality protein maize and five Ethiopian adapted normal maize cultivars. Food Nahrung.

[19] Zarkadas C.G., Hamilton R.I., Yu Z.R., Choi V.K., Khanizadeh S., Rose N.G., Pattison P.L. (2000). Assessment of the protein quality of 15 new northern adapted cultivars of quality protein maize using amino acid analysis. J. Agric. Food Chem..

[20] Sousa R., Recio I., Heimo D., Dubois S., Moughan P.J., Hodgkinson S., Egger L. (2022). In vitro digestibility of dietary proteins and in vitro DIAAS analytical workflow based on the INFOGEST static protocol and its validation with in vivo data. Food Chem..

[21] Brodkorb A., Egger L., Alminger M., Alvito P., Assuncao R., Ballance S., Recio I. (2019). INFOGEST static in vitro simulation of gastrointestinal food digestion. Nat. Protoc..

[22] Gorissen S.H.M., Crombag J.J.R., Senden J.M.G., Waterval W.A.H., Bierau J., Verdijk L.B., van Loon L.J.C. (2018). Protein content and amino acid composition of commercially available plant-based protein isolates. Amino Acids.

[23] FAO (2011). Dietary protein quality evaluation in human nutrition. FAO Food Nutr. Pap..

[24] Gilani G., Tomé D., Moughan P., Burlingame B. (2015).

[25] Cervantes-Pahm S.K., Liu Y., Stein H.H. (2014). Digestible indispensable amino acid score and digestible amino acids in eight cereal grains. Br. J. Nutr..

[26] Rodriguez D.A., Lee S.A., Jones C.K., Htoo J.K., Stein H.H. (2020). Digestibility of amino acids, fiber, and energy by growing pigs, and concentrations of digestible and metabolizable energy in yellow dent corn, hard red winter wheat, and sorghum may be influenced by extrusion. Anim. Feed Sci. Technol..

[27] Addinsoft (2023). https://www.xlstat.com/en.

[28] Mariotti F., Tomé D., Mirand P.P. (2008). Converting nitrogen into protein—beyond 6.25 and Jones' factors. Crit. Rev. Food Sci. Nutr..

[29] Tandzi L.N., Mutengwa C.S., Ngonkeu E., Lm, Woïn N., Gracen V. (2017). Breeding for quality protein maize (QPM) varieties: a review. Agronomy.

[30] Shawa H., van Biljon A., Labuschagne M.T. (2021). Protein quality and quantity of quality protein maize (QPM) and non‐QPM hybrids under optimal and low nitrogen conditions. Cereal Chem..

[31] Souffrant W. (2001). Effect of dietary fibre on ileal digestibility and endogenous nitrogen losses in the pig. Anim. Feed Sci. Technol..

[32] Plascencia A., Bermúdez R., Cervantes M., Corona L., Davila-Ramos H., López-Soto M., Zinn R. (2011). Influence of processing method on comparative digestion of white corn versus conventional steam-flaked yellow dent corn in finishing diets for feedlot cattle. J. Anim. Sci..

[33] Borrás L., Caballero-Rothar N.N., Saenz E., Segui M., Gerde J.A. (2022). Challenges and opportunities of hard endosperm food grade maize sourced from South America to Europe. Eur. J. Agron..

[34] Kaczmarek S., Cowieson A., Józefiak D., Rutkowski A. (2013). Effect of maize endosperm hardness, drying temperature and microbial enzyme supplementation on the performance of broiler chickens. Anim. Prod. Sci..

[35] Muleya M., Young S.D., Bailey E.H. (2021). A stable isotope approach to accurately determine iron and zinc bioaccessibility in cereals and legumes based on a modified INFOGEST static in vitro digestion method. Food Res. Int..

